# The Relationship Between Composite Inflammatory Indices and Dry Eye in Hashimoto’s Disease-Induced Hypothyroid Patients

**DOI:** 10.3390/biomedicines13112675

**Published:** 2025-10-30

**Authors:** Asli Kirmaci Kabakci, Derya Cepni Cakir, Arzu Taskiran Comez

**Affiliations:** Department of Ophthalmology, Prof. Dr. Cemil Tascioglu City Hospital, University of Health Sciences, Istanbul 34384, Turkey

**Keywords:** Hashimoto’s thyroiditis, hypothyroidism, dry eye disease, systemic inflammation, systemic inflammation response index, C-reactive protein-to-albumin ratio, anti-TPO

## Abstract

**Background/Objectives**: Hashimoto’s thyroiditis-induced hypothyroidism (HT–HypoT) is frequently accompanied by ocular surface complaints, but the role of systemic inflammatory markers in dry eye disease (DED) among these patients remains unclear. This study aimed to evaluate the relationship between composite inflammatory indices and the presence and severity of DED in patients with HT–HypoT. **Methods**: This retrospective study included 86 HT–HypoT patients and 43 DED controls without systemic comorbidities. DED diagnosis and severity were assessed using the Ocular Surface Disease Index (OSDI) and objective ocular surface tests. Laboratory parameters and composite inflammatory indices—including neutrophil-to-lymphocyte ratio (NLR), platelet-to-lymphocyte ratio (PLR), systemic immune-inflammation index (SII), systemic inflammation response index (SIRI), C-reactive protein-to-albumin ratio (CAR), and prognostic nutritional index (PNI)—were compared between groups. **Results**: DED was present in 44% of HT–HypoT patients. SIRI and CAR were higher in HT–HypoT patients with DED and increased with severity. Both indices independently predicted the presence and severity of DED and exhibited higher diagnostic performance than other inflammatory indices. **Conclusions**: In patients with HT–HypoT, SIRI and CAR provide additional diagnostic value for identifying the presence and severity of DED beyond that offered by traditional markers. These findings highlight the potential utility of routine blood-derived indices as adjunctive biomarkers in thyroid-related DED.

## 1. Introduction

Hashimoto’s thyroiditis (HT) is the most prevalent autoimmune disorder of the thyroid gland and a leading cause of hypothyroidism in iodine-sufficient populations [1]. A recent systematic review and meta-analysis reported an estimated global prevalence of 7.5% for HT, identified significant regional differences, and found that women have a fourfold greater risk compared to men [2]. Pathophysiologically, HT is characterized by chronic lymphocytic infiltration and fibrosis of the thyroid tissue, accompanied by elevated anti-thyroid antibody levels and gradual glandular destruction [3]. This autoimmune process underlies the development of hypothyroidism (HT–HypoT) and may also have systemic implications beyond the thyroid itself.

Emerging evidence indicates that the inflammatory mechanisms in HT can extend to the ocular surface. In particular, the chronic immune changes that damage the thyroid may also affect exocrine glands like the lacrimal gland, contributing to tear film dysfunction [4]. Additionally, thyroid disorders have been linked to meibomian gland dysfunction, suggesting an evaporative component to dry eye disease (DED) in this patient population [5]. DED is a multifactorial ocular surface disorder characterized by loss of tear film homeostasis and ocular surface inflammation [6]. Thyroid dysfunction, a common endocrine disorder, has a well-recognized association with ocular morbidities [7]. Clinically, DED appears to be a frequent comorbidity in patients with HT–HypoT. A recent study found that more than half of HT patients (54%) had signs of DED, and notably the tear interleukin-6 (IL-6) level was significantly elevated in the HT-DED subgroup (with a concurrent decrease in the anti-inflammatory cytokine IL-10) [4]. Importantly, these findings were observed in euthyroid HT patients without thyroid-associated ophthalmopathy (Graves’ orbitopathy) [4], underscoring that ocular surface involvement can occur in HT independently of overt thyroid eye disease (TED). Moreover, a recent cross-sectional study found that nearly half (47.5%) of DED patients had underlying thyroid disorders [5], underscoring the interrelationship between thyroid imbalances and ocular surface disease. This bidirectional association highlights the importance of investigating inflammatory pathways common to both conditions.

Tear-film hyperosmolarity destabilizes the ocular surface and triggers an inflammatory cascade that recruits innate and adaptive immune cells [8,9]. Infiltrating neutrophils, macrophages, dendritic cells, and T cells release cytokines (e.g., IFN-γ, TNF-α, IL-1 family, IL-17) that injure the epithelium, reduce goblet-cell density, and impair lacrimal function, thereby worsening tear-film instability [10,11,12,13]. Consistent with this, patients with DED exhibit elevated tear cytokines, supporting an inflammation-driven pathogenesis [14,15]. Therefore, DED can be viewed as a chronic ocular surface inflammatory disorder, where local cytokine networks and immune cell infiltration play a pivotal role in disease progression.

Given this inflammatory basis, there is growing interest in systemic inflammatory markers as potential indicators of DED status. In particular, composite inflammatory indices derived from peripheral blood counts have emerged as accessible surrogates of systemic inflammation. The neutrophil-to-lymphocyte ratio (NLR) and the platelet-to-lymphocyte ratio (PLR) are two indicators reflecting the balance between the innate immune response and adaptive immunity, and have been associated with DED in previous studies [16,17,18]. Beyond NLR and PLR, newer markers like the systemic immune-inflammation index (SII) and systemic inflammatory response index (SIRI) integrate multiple blood parameters to provide a more comprehensive measure of inflammation. SII is calculated from neutrophil, lymphocyte, and platelet counts, while SIRI incorporates neutrophil, monocyte, and lymphocyte counts [19]. While limited studies have shown an association between the SII and DED [20,21], the relationship between the SIRI or CAR and DED has not yet been evaluated. In addition, the C-reactive protein/albumin ratio (CAR), a sensitive indicator of systemic inflammatory burden, has drawn attention as a potential biomarker, though it has only been studied in the context of DED among diabetic individuals [22]. To the best of our knowledge, no prior study has conducted a comprehensive assessment of these systemic inflammatory indices in the context of DED among patients with HT–HypoT. This notable absence underscores a critical gap in the current understanding of the interplay between systemic inflammation and ocular surface immunopathology. On the other hand, clinically, these low-cost and reproducible inflammatory indices, which can be easily derived from routine laboratory panels, may be integrated into standard endocrine or ophthalmologic assessments to help prioritize HT-HypoT patients at risk for DED. This approach could facilitate early diagnosis, timely referral, and longitudinal monitoring alongside thyroid function testing.

Based on the above evidence, we hypothesize that DED in HT–HypoT patients is associated with elevated systemic inflammatory indices, reflecting an underlying proinflammatory state that links thyroid dysfunction to ocular surface damage. This study aims to evaluate the role of systemic inflammation in the pathogenesis of DED among patients with HT–HypoT by analyzing composite inflammatory indices, including NLR, PLR, SII, SIRI, CAR and prognostic nutritional index (PNI), and to investigate their potential prognostic value in diagnosis and clinical assessment.

## 2. Materials and Methods

This retrospective, single-center study was conducted in the Department of Ophthalmology at Prof. Dr. Cemil Taşcıoğlu City Hospital between January 2023 and June 2025. The study was designed in accordance with the Declaration of Helsinki. Ethics committee approval was obtained from the Bezmialem University Ethics Committee (Approval number: E-54022451-050.04-202919, Date: 28 July 2025).

### 2.1. Study Design and Population

During the study period, we retrospectively screened consecutive adult patients who attended the ophthalmology clinic and underwent a standardized dry-eye evaluation in routine care. A total of 640 records were assessed for eligibility. Eligible participants for the HT–hypothyroidism group were adults aged 18 years or older with a confirmed diagnosis of HT–HypoT who had undergone evaluation for DED, whereas eligible participants for the control group were individuals without systemic comorbidities who had a documented diagnosis of DED. This control group was selected intentionally to account for the proinflammatory background of DED itself [16,17,21]. By comparing DED patients with HT–HypoT to those with no systemic comorbidities, we aimed to isolate the additional systemic inflammatory burden attributable to autoimmune thyroid disease, beyond the effects of DED alone. Exclusion criteria were: age < 18 years; any ocular disease or history of ocular trauma other than DED (e.g., active ocular infection, uveitis, prior glaucoma surgery, corneal dystrophy); TED or occult TED; thyroid dysfunctions other than HT–HypoT (e.g., hyperthyroidism/Graves); additional systemic comorbidities (including Sjögren syndrome) or active systemic infection; pregnancy; cigarette smoking; contact lens use; and receipt of systemic or ocular anti-inflammatory treatments (including topical corticosteroids) within the previous 3 months; or incomplete/missing medical records. After applying the exclusion criteria, 143 individuals met eligibility (92 with HT–HypoT; 51 without systemic disease for the control pool). To minimize baseline imbalances, we performed propensity score matching on age and sex using nearest-neighbor matching without replacement at a 2:1 ratio (thyroid:control) with a caliper of 0.2 SD of the logit of the propensity score. The final analytic sample comprised 86 patients with HT–HypoT and 43 matched controls.

Clinical and demographic findings of the patients were recorded retrospectively from electronic files. Diagnosis of HT–HypoT was based on elevated TSH with decreased free T4 together with positive anti-TPO and/or anti-Tg antibodies, and was supported by thyroid ultrasonography when available.

### 2.2. Ophthalmic Assessments

Symptoms were assessed using the Ocular Surface Disease Index (OSDI; 0–100) [23]. DED severity was categorized as mild–moderate (OSDI 13–32) and severe (OSDI ≥ 33) [21]. Following this, objective assessments of the ocular surface were performed, comprising noninvasive break-up time (NBUT) measured on a Sirius corneal topographer (Costruzioni Strumenti Oftalmici, Florence, Italy), non-anesthetized Schirmer testing using standard strips (ERC Sağlık, Ankara, Turkey), and corneal fluorescein staining using sodium fluorescein ophthalmic strips (OptiGlo; Wizcure Pharma Pvt. Ltd., Bhiwadi, Rajasthan, India) [6,24]. The following diagnostic parameters were accepted as a patient with DED: an OSDI score ≥ 13 and Schirmer 1 test ≤ 5 mm/5 min or NBUT < 10 s or ocular surface staining score ≥ 1 [6]. Because both eyes were measured, bilateral metrics were summarized at the patient level using a prespecified “worse-eye” rule [25].

### 2.3. Laboratory Measurements

Blood samples were routinely collected from the antecubital vein at approximately 8 a.m., following a minimum of 8 h of overnight fasting, for the assessment of hematological and biochemical parameters. Hematological parameters are analyzed in the same laboratory using the Beckman Coulter AU 5800 (Beckman Coulter, Brea, CA, USA) device. Composite inflammatory indices were calculated as follows: NLR = neutrophil count/lymphocyte count; PLR = platelet count/lymphocyte count; SII = platelet count × neutrophil count/lymphocyte count; SIRI = neutrophil count × monocyte count/lymphocyte count; CAR = CRP/albumin. PNI = 10 × albumin + 0.005 × lymphocyte count. All indices were computed on the day of ocular assessment.

### 2.4. Statistical Analysis

All analyses were performed using SPSS version 24.0 (IBM Corp., Armonk, NY, USA) and R version 4.3.0 (R Foundation for Statistical Computing, Vienna, Austria). Normality of continuous variables was assessed with the Kolmogorov–Smirnov test. Continuous variables were presented as mean ± standard deviation (SD) if normally distributed, or median (interquartile range, IQR) if non-normally distributed; categorical variables were summarized as frequencies and percentages. Between-group comparisons were performed using the one-way ANOVA (post hoc test: Bonferroni test) for normally distributed variables, and the Kruskal–Wallis H test (post hoc test: Dunn test) for non-normally distributed variables. Categorical variables were compared using the Chi-square test or Fisher’s exact test.

Variable selection for laboratory parameters was conducted using least absolute shrinkage and selection operator (LASSO) logistic regression and partial least squares discriminant analysis (PLS-DA) [26,27]. For LASSO, we applied logistic regression with an L1 penalty, and the optimal regularization parameter [log10(C)] was selected via 5-fold stratified cross-validation, optimizing for the area under the receiver operating characteristic curve (AUC-ROC). Standardized coefficient paths were plotted across log10(C) values, and variables with non-zero coefficients at the optimal penalization point were retained for downstream multivariable modeling [27]. In parallel, PLS-DA was conducted to extract latent components that best separated DED subgroups. The optimal number of latent factors in each model was determined using leave-one-out cross-validation procedure, and variable importance in projection (VIP) scores, % explained variance, and classification accuracy were computed [28,29]. VIP scores ≥ 0.8 indicated variable importance in the PLS-DA models [29,30]. To assess discriminative ability, AUC-ROC analysis was performed for selected predictors. AUC values and 95% confidence intervals (CIs) were estimated using nonparametric bootstrap resampling (1000 iterations) [31,32].

Selected variables were then evaluated in multivariable logistic regression models to identify independent predictors of DED. Adjusted odds ratios (ORs) and 95% CIs were reported. AUC-ROC analyses were conducted to evaluate the diagnostic performance of all laboratory parameters in predicting the presence and severity of DED among patients with HT–HypoT. Optimal cutoff points were identified using the Youden index, and the DeLong test was employed to compare ROC curves of independent predictors [33].

A two-tailed *p*-value < 0.05 was considered statistically significant.

## 3. Results

A total of 86 HT–HypoT (mean age 46.3 ± 12.0 years) and 43 DED+ control subjects (mean age 46.7 ± 12.6 years) were included in the analysis. The majority of participants in both groups were female. Thyroid indices confirmed biochemical hypothyroidism in both HT cohorts versus controls. All patients with HT–HypoT were under treatment with levothyroxine. Among patients with HT–HypoT, 60.5% (*n* = 52) had an OSDI ≥13, and 38 of these demonstrated concordant objective ocular-surface findings fulfilling DED criteria. Autoimmune activity was greatest in HT–HypoT/DED+, with Anti-TPO titers 748 (IQR: 403–1070) IU/mL versus 426 (IQR: 245–565) IU/mL in HT–HypoT/DED− and 0.8 (IQR: 0.1–2.40) IU/mL in controls (*p* < 0.001). Compared to the other groups, the HT–HypoT/DED+ group exhibited higher levels of neutrophils, monocytes, CRP, SIRI (Control: 0.7 ± 0.2 vs. HT–HypoT/DED−: 0.7 ± 0.2 vs. HT–HypoT/DED+: 1.0 ± 0.3; *p* < 0.001), and CAR (Control: 0.8 vs. HT–HypoT/DED−: 0.8 vs. HT–HypoT/DED+: 1.2; *p* < 0.001) (Table 1).

In HT–HypoT patients, the mild-to-moderate and severe DED groups exhibited anti-TPO levels compared to the DED− group (HT–HypoT/DED-: 426 vs. mild-to-moderate DED: 731 vs. severe DED: 774; *p* = 0.001). Compared to the other groups, the severe DED group exhibited higher levels of neutrophils, monocytes, CRP, SIRI (HT–HypoT/DED-: 0.7 ± 0.2 vs. mild-to-moderate DED: 0.9 ± 0.2 vs. severe DED: 1.2 ± 0.3; *p* < 0.001), and CAR (HT–HypoT/DED-: 0.8 vs. mild-to-moderate DED: 1.1 vs. severe DED: 1.5; *p* < 0.001) (Table 2).

**Figure 1:**
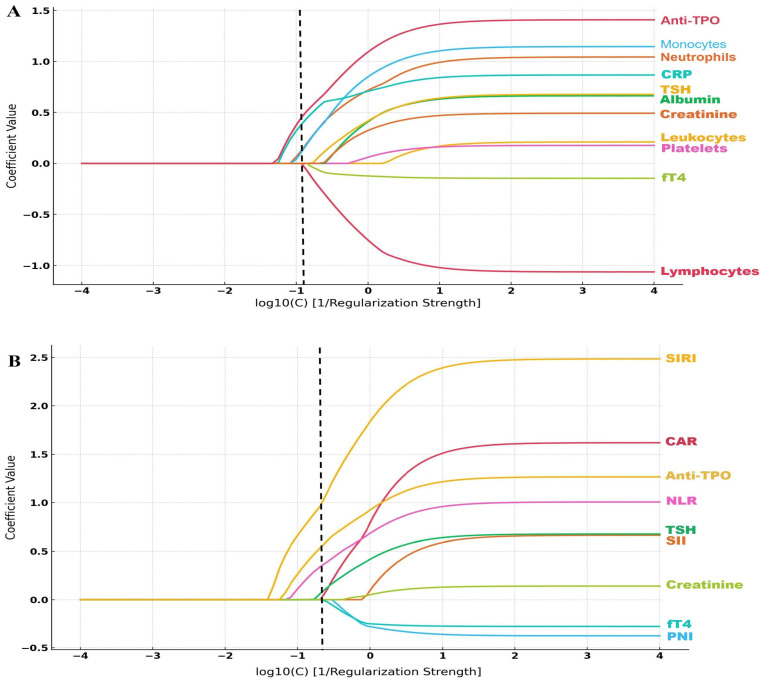
LASSO coefficient paths for the prediction of DED in patients with HT–HypoT. (**A**) Model using only component-level (raw) laboratory parameters. (**B**) Index + core model using a combination of derived systemic inflammatory/nutritional indices and selected core variables. Each curve shows the standardized coefficient of a variable across different log10(C) values, where C is the inverse of regularization strength. As regularization decreases (rightward along the *x*-axis), more variables enter the model with non-zero coefficients. The vertical dashed lines indicate the optimal penalization levels: log10(C) = −0.98 for the component model (AUC = 0.90, 95% CI = 0.86–0.95) and log10(C) = −0.75 for the index + core model (AUC = 0.95, 95% CI = 0.91–0.98), both selected via 5-fold stratified cross-validation and 1000 bootstrap resampling for 95% CI of AUC. At this penalization level, only variables with non-zero coefficients were considered informative and retained for further modeling.

To assess the predictive utility of systemic inflammatory and nutritional parameters in DED among patients with HT–HypoT, we developed two LASSO regression models (Figure 1). The first model (component model) incorporated only raw laboratory variables directly reflecting immune and inflammatory activity (e.g., CRP, monocytes, lymphocytes). The second model (index + core model) integrated derived composite indices (e.g., SIRI, CAR, PNI), which summarize systemic inflammatory responses, along with key disease-related biochemical markers (e.g., Anti-TPO, TSH, T4, creatinine). The coefficient path plots in Figure 1 illustrate the evolution of standardized coefficients across varying regularization strengths, highlighting the most informative features in each model. Notably, Anti-TPO emerged as a consistent predictor across both models. While anti-TPO levels, neutrophil count, monocyte count, and CRP levels showed a positive association with DED in the component model, composite indices such as SIRI and CAR were more prominent in the index + core model.

The comparative performance metrics of PLS-DA models are summarized in Table 3, indicating that both models explained a substantial proportion of outcome variance (70–75%) and demonstrated high discriminatory power (AUC = 0.90–0.95). Among the inflammatory indices, SIRI (VIP: 2.49) and CAR (VIP: 1.56) were found to have the strongest positive associations with the outcome. These findings suggest that while individual inflammatory parameters retain diagnostic value, integration of systemic indices may enhance model performance in capturing complex immuno-inflammatory patterns related to DED. While the component model yielded an accuracy of 82.5% in case classification, the index + core model demonstrated improved performance, correctly classifying 90.2% of cases.

Based on variables selected through both LASSO and PLS-DA analyses, multivariable logistic regression analysis revealed that anti-TPO (OR = 1.03, 95% CI = 1.01–1.05, *p* < 0.001), SIRI (OR = 1.06, 95% CI = 1.03–1.10, *p* < 0.001), and CAR (OR = 1.22, 95% CI = 1.04–1.44, *p* < 0.001) levels independently predicted the presence of DED, while SIRI (OR = 1.05, 95% CI = 1.02–1.10, *p* < 0.001) and CAR (OR = 1.19, 95% CI = 1.05–1.32, *p* < 0.001) were independent predictors of severe DED (Table 4).

In patients with HT–HypoT, specific DED parameters such as the Schirmer test and NIBUT did not show any significant correlation with antibody titers or inflammation/nutrition indices. However, OSDI scores were positively correlated with both SIRI (r = 0.477, *p* < 0.001) and CAR (r = 0.431, *p* < 0.001) levels.

**Figure 2:**
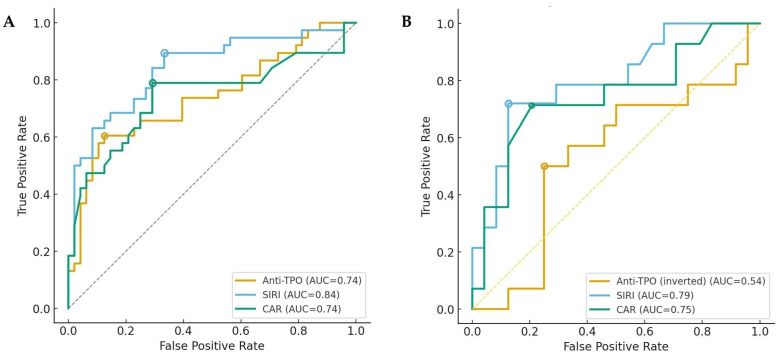
Diagnostic performance of anti-TPO, SIRI, and CAR in predicting the presence (**A**) and severity (**B**) DED in patients with HT–HypoT. Colored circles indicate the optimal thresholds determined by maximizing Youden’s index for each ROC curve.

ROC analysis revealed that SIRI had the highest AUC value in predicting both the presence and severity of DED (AUC: 0.84, 95% CI = 0.74–0.91, Sensitivity: 89.5%, Specificity: 66.7% for DED+; AUC: 0.79, 95% CI = 0.62–0.93, Sensitivity: 72.3%, Specificity: 87.5% for severe DED). CAR and anti-TPO levels demonstrated similar diagnostic performance in predicting DED presence (AUC: 0.75 vs. 0.74). However, CAR showed superior diagnostic performance compared to anti-TPO in predicting DED severity (AUC: 0.75, 95% CI = 0.58–0.90, Sensitivity: 71.4%, Specificity: 79.2% for CRP; AUC: 0.54, 95% CI = 0.39–0.67, Sensitivity: 92.9%, Specificity: 11.9% for anti-TPO) (Figure 2). The diagnostic performance of laboratory parameters and derived indices is presented in Supplementary Materials Tables S1 and S2.

## 4. Discussion

To the best of our knowledge, this is one of the first comprehensive studies to report the association between DED and composite inflammatory markers in patients with HT–HypoT. Neutrophil-based inflammatory indices and the CAR were markedly higher in HT–HypoT patients with DED. Notably, higher SIRI and CAR values correlated with more severe DED manifestations. Furthermore, SIRI demonstrated superior diagnostic performance relative to the other indices, yielding the highest AUC values for identifying the presence of DED as well as for discriminating severe DED. These principal findings suggest that among the various blood-derived inflammatory indices, SIRI and CAR may be the most sensitive indicators of DED risk and severity in patients with HT–HypoT.

Approximately 44% of patients in the HT–HypoT group exhibited DED. Studies have reported a higher prevalence of DED in patients with hypothyroidism compared to euthyroid individuals [34,35]. In a 2025 study of HT patients without orbitopathy, over half exhibited objective DED, and nearly half of these had severe symptoms, yet anti-TPO titers showed no consistent correlation with subjective (OSDI) or objective (Schirmer and NIBUT) measures of DED [4]. In our cohort, antibody or inflammatory indices showed no significant correlation with objective measures, whereas inflammatory indices were positively associated with subjective measures. This highlights the common disconnect between dry eye signs and symptoms [36,37,38]. In fact, prior studies have shown that OSDI scores correlate more strongly with meibography parameters such as gland dropout and meibomian gland loss area, while their correlation with Schirmer or NIBUT is only moderate [35,39]. Moreover, meibomian gland dysfunction has itself been linked to inflammatory burden [40], supporting a mechanistic link between systemic immune activation and subjective symptom burden in autoimmune DED. Population-level evidence also supports this association: a large Taiwanese cohort study showed that patients with autoimmune thyroid disease had a 31% higher hazard of corneal surface damage—including recurrent erosions and persistent epithelial defects—than individuals without thyroid autoimmunity [41].

The altered cytokine milieu observed in HT patients may contribute to this risk by promoting chronic ocular surface inflammation and increasing the susceptibility to DED [42]. In addition, the innate immune profile of the cornea has been linked to ocular surface inflammatory disorders, including DED [43]. These findings may reflect differences in the activation of circulating immune-inflammatory cells in HT patients and an exaggerated inflammatory cascade in the presence of DED. Several studies have reported elevated composite inflammatory indices—such as NLR, PLR, SII, and SIRI—in patients with autoimmune thyroiditis [44,45,46,47,48]. However, the relationship between these indices and DED has not been thoroughly investigated in the HT–HypoT population. Meng et al. reported that NLR was significantly higher in DED patients without comorbidities compared to healthy controls, whereas PLR showed no difference between groups [17]. Another study involving patients without additional systemic diseases reported significantly higher NLR levels in those with DED, while CRP levels showed no significant variation [49]. In another study involving patients with a similar clinical profile, NLR and CRP levels exhibited a comparable pattern, whereas PLR values were reported to be higher in patients with DED [16]. A recent study demonstrated that the SII was significantly higher in non-Sjögren patients with DED compared to healthy individuals [21]. Similarly, in primary Sjögren’s syndrome (an autoimmune condition with severe DED), higher SII values were strongly associated with disease activity and ocular dryness [20]. These findings from the literature corroborate our evidence that systemic inflammatory burden is increased in DED, while also highlighting variability between studies and patient subsets.

HT–HypoT/DED+ patients had higher levels of NLR, SIRI, and CAR, whereas platelet-based indices did not show significant differences. Among all indices, SIRI and CAR were independently associated with both the presence and severity of DED, and demonstrated superior diagnostic performance. This likely reflects the mechanistic relevance of their cellular components. SIRI incorporates neutrophils, monocytes, and lymphocytes—key mediators of innate immunity—whereas PLR and SII depend on platelets, which play a more limited role in inflammatory signaling. Neutrophils from HT patients exhibit increased Neutrophil Extracellular Trap formation (NETosis), which promotes IL-6 production [50], while both neutrophils and monocyte/macrophage activation contribute to IL-6, IL-1β, and TNF-α release [51,52]. These cytokines are prominent in the tear profiles of DED patients [53,54,55], and IL-6 and MMP-9, in particular, is elevated in HT/DED+ tears [4]. IL-6 also stimulates hepatic CRP synthesis, thereby contributing to elevated CAR levels [56]. This pathway provides a mechanistic link between thyroid autoimmunity and the systemic inflammatory burden that may contribute to ocular surface pathology, as captured by elevated SIRI and CAR. In contrast, platelets themselves are not major producers or targets of IL-6 in immune regulation. The IL-6–platelet link is chiefly indirect: IL-6 elevates platelet counts via the liver and bone marrow axis, but platelets do not significantly feed back on IL-6 [57]. This mechanism may underlie the absence of a significant relationship between platelet and platelet-based indices and DED presence or severity. These findings were further supported by multivariable feature selection (LASSO and PLS-DA analyses), where neutrophil- and monocyte-driven indices emerged as dominant predictors, while platelet-related metrics were not prioritized.

This study highlights the clinical utility of routinely available blood-derived inflammatory indices in patients with HT–HypoT. The identification of SIRI and CAR as strong independent predictors of both the presence and severity of DED suggests that these indices may serve as adjunctive, non-invasive biomarkers to complement ophthalmic examinations. Given their low cost, accessibility, and repeatability, SIRI and CAR offer promising value—particularly in resource-limited settings or in primary care, where specialized ocular diagnostics may not be readily available. These markers could serve as blood-based surrogates for tear cytokine profiling, aiding in risk stratification without requiring specialized equipment. Practically, HT–HypoT patients with elevated SIRI and/or CAR can be triaged for earlier ophthalmology referral, tear-film testing, and initiation/intensification of ocular-surface therapy. Integration into the electronic health record (EHR) as a simple risk rule (e.g., OSDI ≥ 13 plus elevated SIRI or CAR → “eye referral” alert) or an easy-to-use nomogram (SIRI + CAR + OSDI ± NIBUT) could streamline interdisciplinary care. Incorporating such indices into routine endocrine or ophthalmologic evaluation could improve risk stratification, early detection, and monitoring of DED in HT patients, especially those with high anti-TPO titers. This may facilitate earlier initiation of targeted interventions, enhance interdisciplinary collaboration between endocrinologists and ophthalmologists, and ultimately improve patient quality of life.

### Limitations

Our study has several limitations that warrant consideration. First, its retrospective design introduces the possibility of selection bias and limits the ability to establish causal relationships. Second, the study was single-center, which may constrain generalizability and invites selection bias. Therefore, prospective, multicenter validation studies are required to confirm these results and assess generalizability across diverse populations. Third, cytokine measurements (e.g., IL-6, IL-10, MMP-9) were not performed, which could have provided additional mechanistic insights linking systemic and local ocular inflammation. Integration of systemic inflammatory indices with tear cytokine profiling and ocular surface biomarkers may help elucidate mechanistic pathways linking thyroid autoimmunity and ocular inflammation. Fourth, we classified severity by OSDI alone for the primary analysis; while aligned with guidelines, severity defined by combined symptom–sign algorithms may offer additional granularity in future work. Longitudinal follow-up studies should evaluate whether SIRI and CAR levels fluctuate in parallel with DED progression or response to therapy, which would strengthen their prognostic utility. Finally, comparative research across other autoimmune diseases may clarify whether these indices are disease-specific or represent general markers of systemic inflammatory activity in ocular surface disease.

## 5. Conclusions

This study demonstrates that systemic immune-inflammatory indices, particularly SIRI and CAR, independently predict the presence and severity of DED in patients with HT–HypoT. Alongside traditional markers such as anti-TPO, these indices capture the complex interplay of systemic autoimmunity and ocular surface inflammation. These easily obtainable indices offer practical, scalable tools to support screening and risk stratification in endocrine and primary-care settings, enabling earlier ophthalmic evaluation and targeted management.

## Figures and Tables

**Table 1:** Demographic and laboratory findings of study population.

Variables	Control Group (*n* = 43)	Hypothyroidism HT		*p*-Value
		Without DED (*n* = 48)	With DED (*n* = 38)	
Age, years	46.7 ± 12.6	45.4 ± 9.0	47.4 ± 15.1	0.734
Gender, *n* (%)				
Female	32 (74.4)	37 (77.1)	29 (76.3)	0.999
Male	11 (25.6)	11 (22.9)	9 (23.7)	
Duration of illness, years	-	2 (1–4)	3 (1–4)	0.846
Schirmer test, mm	8 (5–15)	**15 (8–30)**	7 (4–15)	<0.001 *
NIBUT, s	7 (5–9)	**12 (5–20)**	5 (4–8)	0.002 *
OSDI	17 (15–20)	**8 (3–14)**	**28 (20–38)**	<0.001 *
Laboratory findings				
fT4, ng/dL	**1.0 ± 0.2**	0.5 ± 0.1	0.4 ± 0.1	<0.001 *
TSH, mIU/L	**1.9 (1.5–3)**	8.9 (7.0–15.8)	10.7 (7.9–17.6)	<0.001 *
Anti-TPO, IU/mL	**0.8 (0.1–2.40)**	**426 (245–565)**	**748 (403–1070)**	<0.001 *
Leukocytes, ×10^3^ µL	7.0 ± 1.6	7.4 ± 1.3	7.5 ± 1.6	0.321
Lymphocytes, ×10^3^ µL	2.6 ± 0.8	2.5 ± 0.5	2.3 ± 0.4	0.047 *
Neutrophils, ×10^3^ µL	3.8 ± 0.9	4.0 ± 0.6	**4.4 ± 1.0**	0.001 *
Monocytes, ×10^3^ µL	0.4 ± 0.1	0.4 ± 0.1	**0.5 ± 0.1**	0.028 *
Platelets, ×10^3^ µL	265.5 ± 54.9	262.3 ± 56.4	278.8 ± 50.1	0.858
Albumin, g/dL	4.3 ± 0.3	4.4 ± 0.4	4.4 ± 0.5	0.486
CRP, mg/dL	3.5 (2.5–5.5)	3.5 (2.5–5.3)	**5.3 (4.6–6.7)**	0.001 *
Creatinine, mg/dL	0.7 ± 0.1	0.7 ± 0.1	0.7 ± 0.2	0.461
NLR	1.6 ± 0.4	1.7 ± 0.4	**2.0 ± 0.4**	<0.001 *
PLR	117.3 ± 40.7	108.2 ± 36.6	114.3 ± 29.7	0.475
SII	429.5 ± 140.4	444.6 ± 125.2	482.9 ± 143.2	0.198
SIRI	0.7 ± 0.2	0.7 ± 0.2	**1.0 ± 0.3**	<0.001 *
CAR	0.8 (0.6–1.2)	0.8 (0.6–1.1)	**1.2 (1.0–1.7)**	<0.001 *
PNI	55.4 ± 4.6	56.5 ± 3.2	55.2 ± 4.5	0.257

Data are mean ± standard deviation or median (IQR), or number (%). * *p* < 0.05 indicates statistical significance. Bold values represent intergroup differences that reached statistical significance. Abbreviations: Anti-TPO, anti-thyroid peroxidase antibody; CAR, C-reactive protein-to-albumin ratio; CRP, C-reactive protein; DED, dry eye disease; fT4, free thyroxine (Free T4); HT, Hashimoto thyroiditis; NIBUT, non-invasive tear break-up time; NLR, neutrophil-to-lymphocyte ratio; OSDI, Ocular Surface Disease Index; PLR, platelet-to-lymphocyte ratio; PNI, prognostic nutritional index; SII, systemic immune-inflammation index; SIRI, systemic inflammatory response index; TSH, thyroid-stimulating hormone.

**Table 2:** Demographic and laboratory findings by severity of DED.

Variables	HT–HypoT			*p*-Value
	Without DED (*n* = 48)	Mild to Moderate DED (*n* = 24)	Severe DED (*n* = 14)	
Age, years	45.4 ± 9.0	45.3 ± 16.2	51.1 ± 12.8	0.258
Gender, *n* (%)				
Female	37 (77.1)	18 (75.0)	11 (78.6)	0.999
Male	11 (22.9)	6 (25.0)	3 (21.4)	
Duration of illness, years	2 (1–4)	2.5 (1–4)	3 (1–4)	0.742
Schirmer test, mm	**15 (8–30)**	9 (4–15)	8 (4–14)	0.005 *
NIBUT, s	**12 (5–20)**	6 (4–9)	5 (3–8)	0.003 *
OSDI	**8 (3–14)**	**22 (17–28)**	**43 (34–65)**	<0.001 *
Laboratory findings				
fT4, ng/dL	0.5 ± 0.1	0.4 ± 0.1	0.4 ± 0.1	0.901
TSH, mIU/L	8.9 (7.0–15.8)	11.4 (7.9–17.6)	12.1 (8.8–23.4)	0.238
Anti-TPO, IU/mL	**426 (245–565)**	731 (343–1066)	774 (403–1097)	0.001 *
Leukocytes, ×10^3^ µL	7.4 ± 1.3	7.5 ± 1.7	7.3 ± 1.3	0.899
Lymphocytes, ×10^3^ µL	2.5 ± 0.8	2.3 ± 0.7	2.3 ± 0.5	0.207
Neutrophils, ×10^3^ µL	4.0 ± 0.6	4.2 ± 1.0	**4.6 ± 0.9**	<0.001 *
Monocytes, ×10^3^ µL	**0.4 ± 0.1**	0.5 ± 0.2	0.5 ± 0.1	0.001 *
Platelets, ×10^3^ µL	262.3 ± 56.4	270.0 ± 59.6	279.2 ± 42.7	0.192
Albumine, g/dL	4.4 ± 0.4	4.4 ± 0.3	4.5 ± 0.7	0.440
Creatinin, mg/dL	0.7 ± 0.1	0.7 ± 0.2	0.7 ± 0.1	0.364
CRP, mg/dL	3.5 (2.5–5.3)	5.0 (3.2–5.4)	**6.7 (6.1–9.3)**	<0.001 *
NLR	**1.7 ± 0.4**	**1.9 ± 0.5**	**2.3 ± 0.5**	<0.001 *
PLR	108.2 ± 36.6	112.7 ± 34.5	118.5 ± 19.2	0.659
SII	444.6 ± 125.2	471.1 ± 164.4	503.2 ± 98.2	0.331
SIRI	**0.7 ± 0.2**	**0.9 ± 0.2**	**1.2 ± 0.3**	<0.001 *
CAR	**0.8 (0.6–1.1)**	**1.1 (0.7–1.4)**	**1.5 (1.2–1.7)**	<0.001 *
PNI	56.5 ± 3.2	55.4 ± 5.1	54.8 ± 2.3	0.270

Data are mean ± standard deviation or median (IQR), or number (%). * *p* < 0.05 indicates statistical significance. Bold values represent intergroup differences that reached statistical significance. Abbreviations: Anti-TPO, anti-thyroid peroxidase antibody; CAR, C-reactive protein-to-albumin ratio; CRP, C-reactive protein; DED, dry eye disease; fT4, free thyroxine (Free T4); HT–HypoT, Hashimoto’s thyroiditis-induced hypothyroidism; NIBUT, non-invasive tear break-up time; NLR, neutrophil-to-lymphocyte ratio; OSDI, Ocular Surface Disease Index; PLR, platelet-to-lymphocyte ratio; PNI, prognostic nutritional index; SII, systemic immune-inflammation index; SIRI, systemic inflammatory response index; TSH, thyroid-stimulating hormone.

**Table 3:** Inflammatory markers related to DED in patients with HT–HypoT as identified by PLS-DA analysis.

Characteristics	Component Model			Index + Core Model		
% variation explained by latent factors						
For predictor variables (inflammatory mediators)	0.92			0.95		
For outcome variables (DED)	0.70			0.75		
Number of used latent factors	1			1		
AUC (95% CI)	0.90 (0.85–0.95)			0.95 (0.90–0.99)		
Number of correctly classified (95% CI)	82.5% (77–88%)			90.2% (85–96%)		
*p*-value	<0.001			<0.001		
	**Factor**	**VIP**	**+/−**	**Factor**	**VIP**	**+/−**
Top inflammatory markers responsible for outcome	Anti-TPO	1.48	+	Anti-TPO	1.25	+
	Neutrophils	1.10	+	SIRI	2.49	+
	Monocytes	1.12	+	CAR	1.56	+
	CRP	0.85	+			

Age, gender, and duration of illness were adjusted in all analyses. *p*-values are for the associations between outcome and latent factors. Model performance was evaluated using leave-one-out cross-validation procedure and 1000 bootstrap resampling for 95% CI of AUC. VIP scores ≥ 0.8 indicated variable importance in the models. Abbreviations: see Table 1, VIP, variance importance in projection; +/−, positive/negative association.

**Table 4:** Independent predictors of presence and severe DED in patients with HT–HypoT.

Variables	Univariable Regression		Multivariable Regression	
	OR (95% CI)	*p*	OR (95% CI)	*p*
DED+ vs. DED−				
Anti-TPO	1.03 (1.01–1.05)	<0.001 *	1.03 (1.01–1.05)	<0.001 *
Lymphocytes	0.57 (0.30–0.98)	0.047 *	–	–
Neutrophils	2.34 (1.24–4.40)	0.009 *	–	–
Monocytes	1.07 (1.01–1.13)	0.015 *	–	–
CRP	1.63 (1.25–2.12)	<0.001 *	–	–
NLR	1.03 (1.01–1.06)	<0.001 *	–	–
SIRI	1.07 (1.04–1.10)	<0.001 *	1.06 (1.03–1.10)	<0.001 *
CAR	1.26 (1.11–1.43)	<0.001 *	1.22 (1.04–1.44)	<0.001 *
			Nagelkerke R^2^ = 0.66	
Severe DED vs. mild to moderate DED				
Anti-TPO	1.01 (0.99–1.02)	0.632	–	-
Neutrophils	3.21 (1.12–9.20)	0.030 *	–	-
Monocytes	1.02 (1.01–1.05)	0.022 *	–	-
CRP	1.28 (1.02–1.71)	0.025 *	–	-
NLR	1.19 (1.01–1.40)	0.038 *	–	-
SIRI	1.05 (1.02–1.09)	<0.001 *	1.05 (1.02–1.10)	<0.001 *
CAR	1.16 (1.04–1.27)	<0.001 *	1.19 (1.05–1.32)	<0.001 *
			Nagelkerke R^2^ = 0.51	

Age, gender, duration of illness, TSH, and fT4 were adjusted in all analyses. * *p* < 0.05 indicates statistical significance. Abbreviations: Anti-TPO, anti-thyroid peroxidase antibody; CAR, C-reactive protein-to-albumin ratio; CRP, C-reactive protein; DED, dry eye disease; fT4, free thyroxine (Free T4); HT–HypoT, Hashimoto’s thyroiditis-induced hypothyroidism; NLR, neutrophil-to-lymphocyte ratio; SIRI, systemic inflammatory response index.

## Data Availability

The data that support the findings of this study are available on request from the corresponding author due to privacy and ethical reasons.

## Supplementary Materials

The following supporting information can be downloaded at: https://www.mdpi.com/article/10.3390/biomedicines13112675/s1, Table S1: Diagnostic performance of potential risk factors in predicting the presence of dry eye in patients with Hashimoto’s thyroiditis–induced hypothyroidism; Table S2: Diagnostic performance of potential risk factors in predicting the severe dry eye in patients with Hashimoto’s thyroiditis–induced hypothyroidism.
