# Supplementary material for: The Relationship Between Composite Inflammatory Indices and Dry Eye in Hashimoto’s Disease-Induced Hypothyroid Patients
Source: Biomedicines. 2025 Oct 30;13(11):2675. doi: 10.3390/biomedicines13112675 (PMC12650015; doi:10.3390/biomedicines13112675)
Supplement: Supplementary file 1 [file biomedicines-13-02675-s001.zip › biomedicines-3909777-supplementary.pdf]

Supplementary Table S1. Diagnostic performance of potential risk factors in predicting the presence of dry eye in patients with Hashimoto's thyroiditis–induced hypothyroidism.

Variables	AUC (95% CI)	Sensitivity (95% CI)	Specificity (95% CI)	Cutoff Value	*p*-Value
fT4	0.58 (0.45–0.70)	5.3 (0.0–92.3)	100.0 (15.4–100.0)	<0.55	0.120
TSH	0.59 (0.47–0.71)	52.6 (26.2–97.4)	70.8 (20.9–97.5)	>10.7	0.141
anti-TPO	0.74 (0.63–0.85)	60.5 (43.9–78.4)	87.5 (73.1–98.0)	>694.0	<0.001 *
Leukocytes	0.51 (0.39–0.64)	47.4 (8.4–84.7)	70.8 (27.5–100.0)	>7.6	0.324
Lymphocytes	0.41 (0.28–0.52)	89.5 (18.4–100.0)	22.9 (5.4–90.2)	<1.8	0.159
Neutrophils	0.68 (0.56–0.78)	36.8 (26.5–86.6)	97.9 (52.0–100.0)	>4.8	0.001 *
Monocytes	0.68 (0.54–0.79)	71.1 (27.7–85.4)	64.6 (52.6–99.0)	>0.48	0.023 *
Platelets	0.50 (0.39–0.63)	57.9 (41.6–95.9)	60.4 (19.6–80.2)	>254.0	0.979
CRP	0.71 (0.59–0.82)	76.3 (38.5–89.1)	70.8 (60.0–100.0)	>4.6	<0.001 *
Albumin	0.57 (0.42–0.68)	65.8 (16.2–80.0)	60.4 (47.6–98.1)	>4.3	0.392
Creatinine	0.60 (0.47–0.72)	15.8 (6.2–49.2)	100.0 (72.7–100.0)	>0.9	0.215
NLR	0.73 (0.62–0.84)	71.1 (41.8–90.0)	75.0 (55.6–100.0)	>1.9	<0.001 *
PLR	0.59 (0.46–0.68)	52.6 (37.4–86.6)	81.2 (37.0–93.7)	>119.8	0.485
SII	0.59 (0.47–0.70)	63.2 (24.4–91.5)	60.4 (28.9–95.7)	>437.0	0.151
SIRI	0.84 (0.74–0.91)	89.5 (59.2–97.1)	66.7 (61.2–97.9)	>0.80	<0.001 *
CAR	0.74 (0.63–0.85)	78.9 (43.2–90.4)	70.8 (60.7–97.9)	>1.0	<0.001 *
PNI	0.60 (0.47–0.72)	31.6 (0.0–46.2)	83.3 (74.5–100.0)	<58.050	0.242

* $p < 0.05$ indicates statistical significance. Abbreviations: AUC, area under the curve; AntiTPO, anti-thyroid peroxidase antibody; CAR, C-reactive protein-to-albumin ratio; CI, confidence interval; CRP, C-reactive protein; DED, dry eye disease; fT4, free thyroxine (Free T4); HT, Hashimoto thyroiditis; NIBUT, non-invasive tear break-up time; NLR, neutrophil-to-lymphocyte ratio; OSDI, Ocular Surface Disease Index; PLR, platelet-to-lymphocyte ratio; PNI, prognostic nutritional index; SII, systemic immune-inflammation index; SIRI, systemic inflammatory response index; TSH, thyroid-stimulating hormone.

Supplementary Table S2. Diagnostic performance of potential risk factors in predicting the severe dry eye in patients with Hashimoto's thyroiditis–induced hypothyroidism.

Variables	AUC (95% CI)	Sensitivity (95% CI)	Specificity (95% CI)	Cutoff Value	*p*-Value
T4	0.50 (0.29–0.71)	92.9 (20.0–100.0)	33.3 (15.2–100.0)	<0.38	0.976
TSH	0.58 (0.39–0.67)	100.0 (11.1–100.0)	16.7 (5.4–100.0)	>6.4	0.418
AntiTPO	0.54 (0.37–0.70)	50.0 (36.7–65.2)	75.0 (53.3–90.2)	>541.2	0.714
Leukocytes	0.53 (0.32–0.76)	78.6 (7.7–100.0)	37.5 (26.9–100.0)	>6.7	0.740
Lymphocytes	0.51 (0.32–0.78)	21.4 (3.0–100.0)	91.7 (12.2–100.0)	<3.12	0.903
Neutrophils	0.77 (0.61–0.91)	78.6 (63.0–100.0)	79.2 (38.1–92.2)	>4.7	0.001 *
Monocytes	0.76 (0.57–0.92)	64.3 (35.8–100.0)	83.3 (42.1–100.0)	>0.5	0.002 *
Platelets	0.68 (0.51–0.83)	85.7 (66.7–100.0)	62.5 (33.3–84.0)	>261.0	0.158
CRP	0.69 (0.52–0.85)	92.9 (46.4–100.0)	41.7 (32.7–91.3)	>5.0	0.045 *
Albumin	0.66 (0.43–0.88)	21.4 (0.0–50.0)	91.7 (78.8–100.0)	>5.4	0.079
Creatinine	0.53 (0.35–0.70)	78.6 (50.0–100.0)	37.5 (7.3–81.0)	>0.6	0.772
NLR	0.69 (0.50–0.86)	85.7 (29.2–100.0)	50.0 (27.4–100.0)	>2.0	0.034 *
PLR	0.56 (0.38–0.75)	71.4 (40.6–100.0)	50.0 (10.7–90.2)	>114.4	0.516
SII	0.59 (0.39–0.77)	92.9 (54.7–100.0)	37.5 (19.2–82.0)	>390.5	0.370
SIRI	0.79 (0.62–0.93)	72.3 (61.0–100.0)	87.5 (45.2–100.0)	>1.1	<0.001 *
CAR	0.75 (0.58–0.90)	71.4 (40.8–93.8)	79.2 (49.6–100.0)	>1.5	0.005 *
PNI	0.54 (0.34–0.72)	92.9 (20.7–100.0)	25.0 (8.7–95.9)	<52.3	0.691

* $p < 0.05$ indicates statistical significance. Abbreviations: AUC, area under the curve; AntiTPO, anti-thyroid peroxidase antibody; CAR, C-reactive protein-to-albumin ratio; CI, confidence interval; CRP, C-reactive protein; DED, dry eye disease; fT4, free thyroxine (Free T4); HT, Hashimoto thyroiditis; NIBUT, non-invasive tear break-up time; NLR, neutrophil-to-lymphocyte ratio; OSDI, Ocular Surface Disease Index; PLR, platelet-to-lymphocyte ratio; PNI, prognostic nutritional index; SII, systemic immune-inflammation index; SIRI, systemic inflammatory response index; TSH, thyroid-stimulating hormone.
